# Supplementary material for: Four chromosome scale genomes and a pan-genome annotation to accelerate pecan tree breeding
Source: Nat Commun. 2021 Jul 5;12:4125. doi: 10.1038/s41467-021-24328-w (PMC8257795; doi:10.1038/s41467-021-24328-w)
Supplement: Supplementary file 1 — Supplementary Information [file 41467_2021_24328_MOESM1_ESM.pdf]

Four chromosome scale genomes and a pan-genome annotation to accelerate pecan tree breeding

Lovell *et al.*

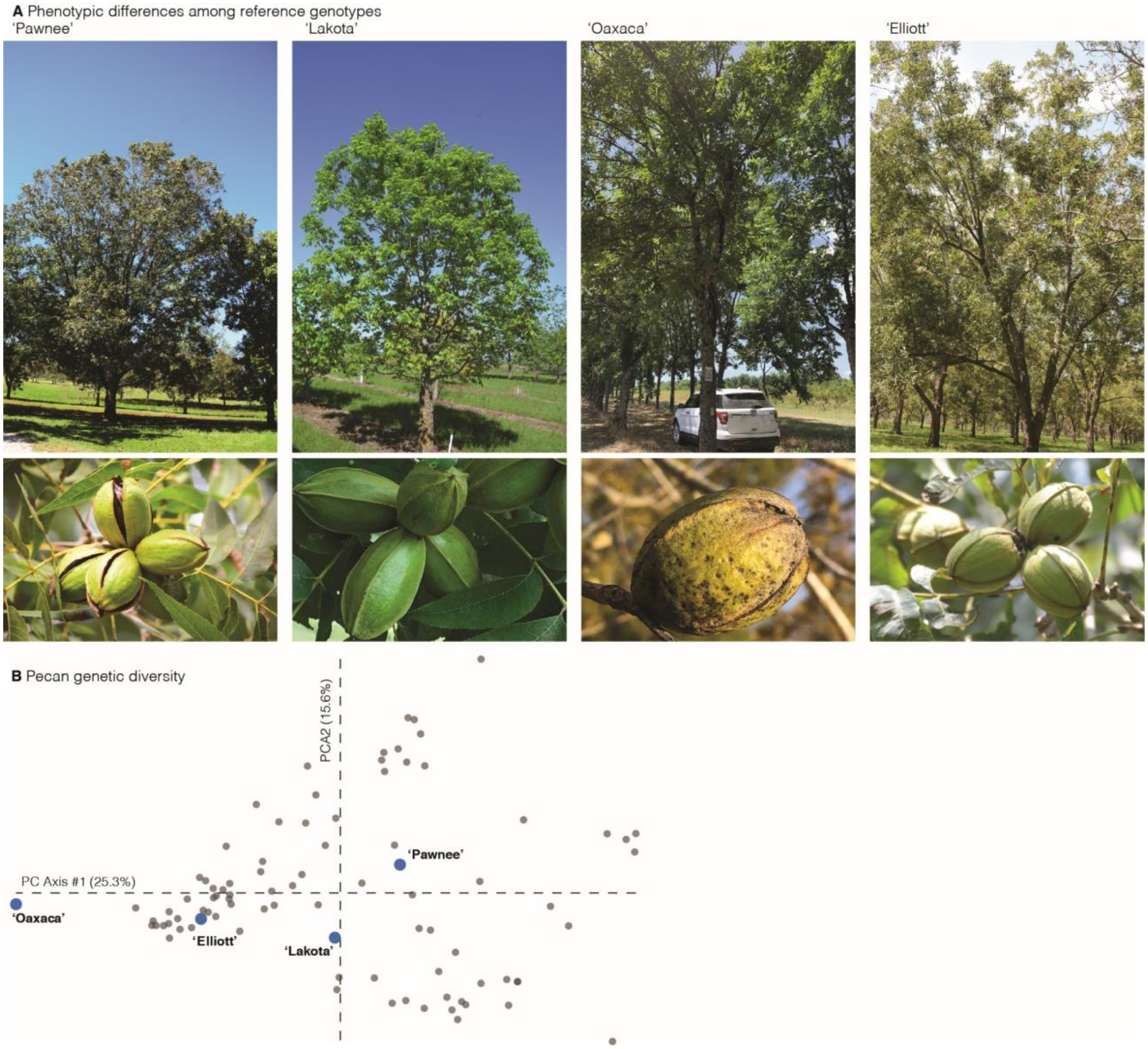

Supplementary Figure 1. Morphology and genetic diversity of reference pecan genotypes. **a**, Field photos of the trees and nut clusters taken just before maturity for the four reference genomes (plant locations): 'Pawnee' (Somerville, TX, USA, 26-October 2017), 'Lakota' (Somerville, TX, USA, 15-April 2016), 'Oaxaca' (Byron, GA, USA, 17-July 2019) and 'Elliott' (Somerville, TX, USA, 23-Sept 2017). **b**, Genetic diversity of a set of 108 pecan genotypes highlighting the four reference genotypes in principal component (PC Axis, PCA) space. Source data underlying Supplementary Figure 1b are provided as a Source Data file.

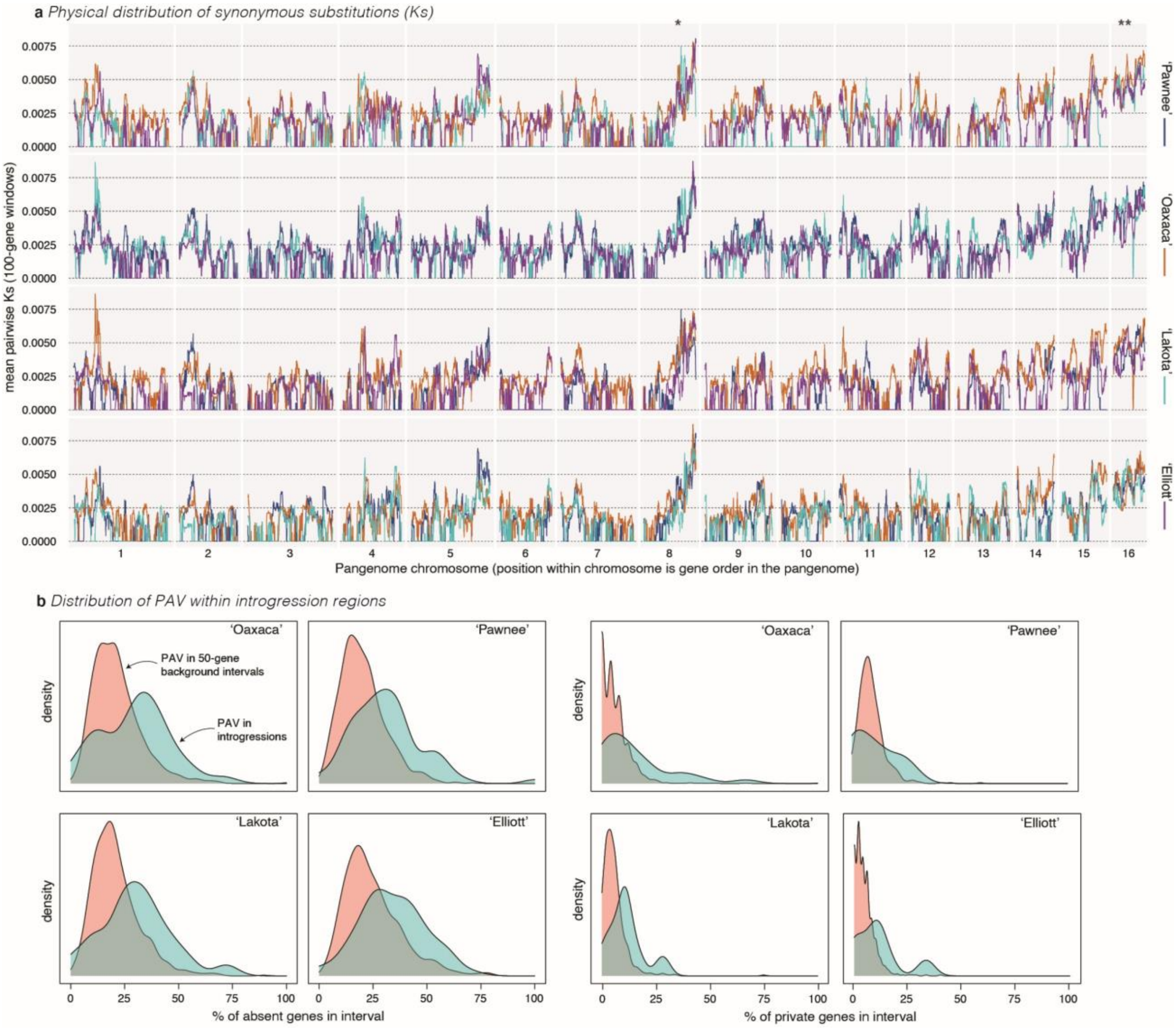

Supplementary Figure 2. Coding and presence-absence variation across the genome and within introgressions. a, Synonymous substitution rate (Ks) was calculated for all 21,196 single copy orthogroups; mean Ks was calculated for each pairwise combination of genomes along 99-gene overlapping 100-gene intervals across the genome. Intervals marked in Fig. 2d are labeled with */** here. **b**, Presence absence variation (PAV) within each genome was extracted from the pan-genome and binned into two groups that may be of significance for evolutionarily independent introgressions: gene models private to a single genome, or genes that were absent in a genome. Mean values were calculated for each of the introgression intervals (Fig. 2b) and all non-overlapping 50-gene intervals that do not include an introgression. Combined, the elevated values of private and absent genes in introgression regions validated the existence and genomic impact of introgressions across the genome. Source data are provided as a Source Data file.

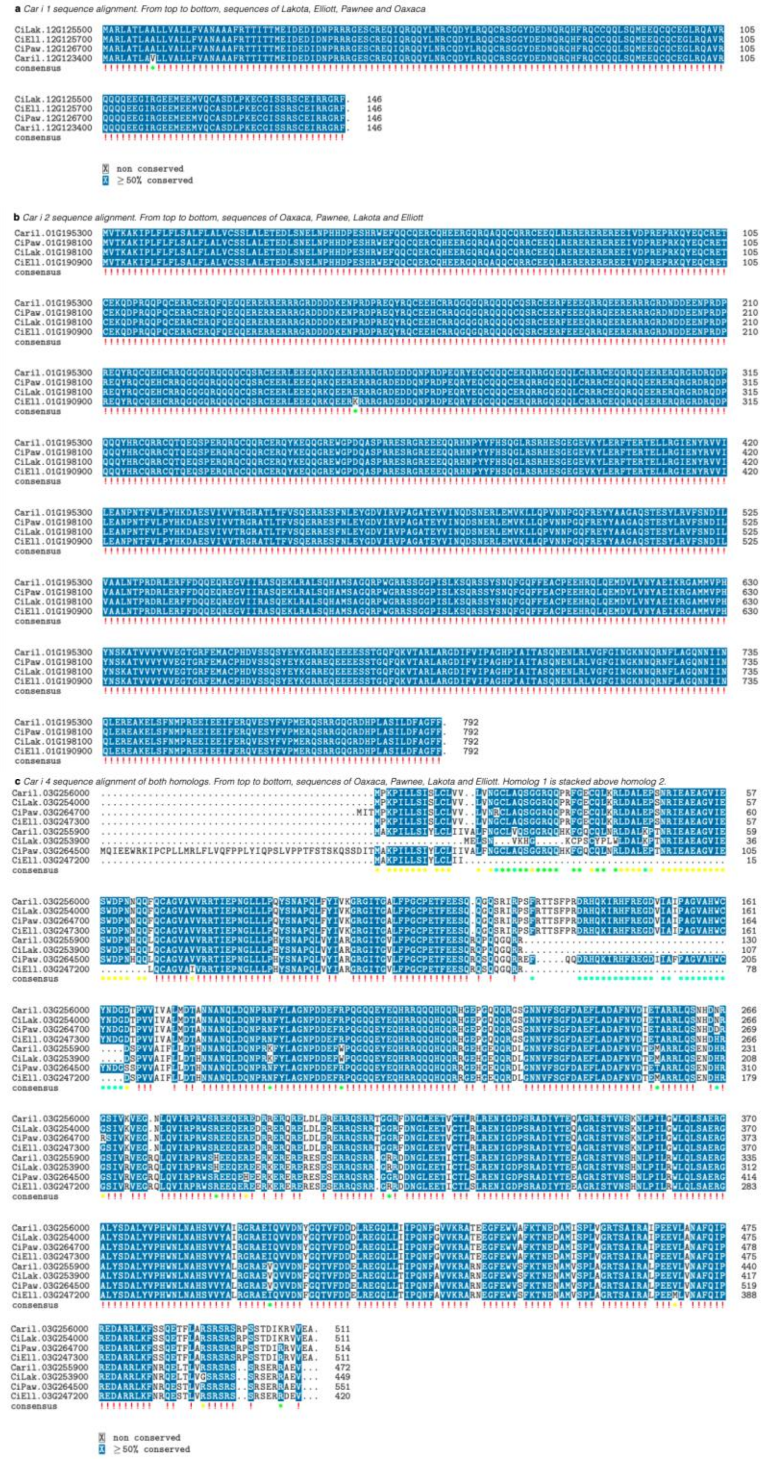

Supplementary Figure 3. Annotated alignments of the three focal allergen protein coding genes. Car i 1 (**a**), Car i 2 (**b**) and Car i 4 (**c**). While Car i 1-2 are single copy, there are two homologs of Car i 4 present in all four genomes. The two copies are included in the same alignment in panel c to show the differences between and within homologs. In all panels, alignments are presented in 105 peptide lines annotated with the gene IDs (left) and position of last base (right). Along the footer of each line is an indicator of whether sites are monomorphic ('!') or polymorphic ('*'), or no annotation if no major allele.

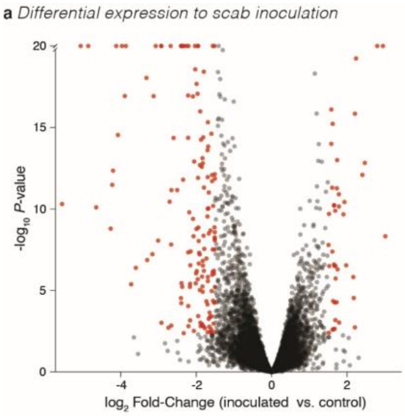

b *Phenotypic confirmation of inoculation*

Treatment	plant ID	n. leaflets	Mean % hyphae*
diH2O (control)**	5	5	0
diH2O (control)**	12	5	0
diH2O (control)**	19	5	0
De-Tif-11	61	3	30.2
De-Tif-11	47	5	64.2
De-Tif-11	54	5	72.8

**Mean calculated across leaflets as the % of conidia with subcuticular hyphae.*

***No V. effusa conidia were observed on any of the control inoculation samples.*

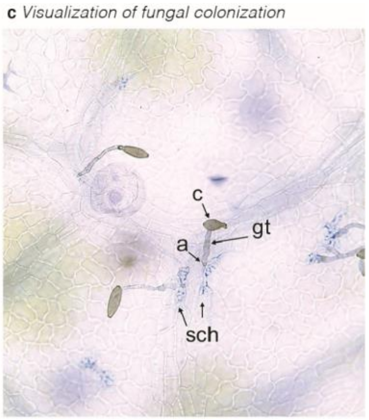

Supplementary Figure 4. Expression and phenotypic response to fungal inoculation. a, Differential expression between control (diH2O) and inoculated treatments of a virulent strain of *V. effusa* (De-Tif-11) in the 'Desirable' susceptible cultivar. Significantly differentially expressed genes are shaded in red; *P*-values < 1×10-20 were thresholded to that value for visualization purposes. This experiment was conducted once at a single time point. **b**, Fungal infection was not observed in the control treatment, but was highly present in all samples of the inoculated treatment. **c**, light microscope image (400×) of fungal colonization: a = appressorium, c = conidium, gt = germ tube, sch = subcuticular hyphae. Raw data associated with Supplementary Figure 4a can be found in Supplementary Data 3.

Supplementary Table 1. Library metadata for genome annotation RNA-sequencing experiments.

Lib*	Genome	Sample	nRaw**	nPrep	nAssign	nNofeat	nAmb	nUnmap	nMulti	Bioproject***	SRA BioID
IKDJ	Oaxaca	Airlayerroots_1	31595167	30689855	21889912	852409	160053	5960676	1826805	PRJNA680537	SRR13411590
IKDP	Oaxaca	Catkin_1	20354464	20007417	12592282	833045	121267	4812867	1647956	PRJNA680537	SRR13411591
IKDU	Oaxaca	Dormantbud_1	31991044	31176496	22129664	1651151	208992	6721732	464957	PRJNA680537	SRR13411592
IKDZ	Oaxaca	Expandingleaf_1	31293011	30591221	22597770	1302362	175107	5815366	700616	PRJNA680537	SRR13411593
IKED	Oaxaca	Pistillateflower_1	33951296	33227504	24247412	1569756	149489	5904165	1356682	PRJNA680537	SRR13411595
IKEH	Oaxaca	Swellingbud_1	38233085	37449807	27391719	1716584	198143	7363861	779500	PRJNA680537	SRR13411594
IKDK	Elliott	Expandingleaf_1	36095993	35529055	24499427	1484888	275275	8545903	723562	PRJNA680537	SRR13413659
IKDQ	Elliott	Swellingbud_3	30125676	29537741	20443418	1040238	258331	7201530	594224	PRJNA680537	SRR13413660
IKDW	Elliott	Swellingbud_1	39335301	38475648	26598709	1537635	310354	9274259	754691	PRJNA680537	SRR13413661
IKEL	Elliott	Dormantbud_1	33299467	32150326	23201642	1418233	179342	6805082	546027	PRJNA680537	SRR13413662
IKEQ	Elliott	Expandingleaf_2	32900656	32389745	23718103	1175931	149191	6762986	583534	PRJNA680537	SRR13413663
IKEU	Elliott	Swellingbud_2	34841228	34333563	24768532	1356877	181699	7399093	627362	PRJNA680537	SRR13413664
IKEZ	Elliott	Dormantbud_2	37472079	36944722	26851345	1635879	181200	7644937	631361	PRJNA680537	SRR13413665
IKFD	Elliott	Dormantbud_3	28023720	27690668	19942968	1246397	155960	5822356	522987	PRJNA680537	SRR13413666
IKFH	Elliott	Expandingleaf_3	29565351	29194307	20334623	1285669	171462	6810776	591777	PRJNA680537	SRR13413667
IKDL	Lakota	Pistillateflower_1	30930962	30597206	21982479	1584763	256411	5272765	1500788	PRJNA680537	SRR13419224
IKDR	Lakota	Swellingbud_2	30064474	29762491	21457850	1204957	223317	5786197	1090170	PRJNA680537	SRR13419225
IKEA	Lakota	Catkin_1	27219338	26642794	18261603	1392318	192338	5454457	1342078	PRJNA680537	SRR13419226
IKEE	Lakota	Dormantbud_1	30212483	29635989	20160259	2551427	177993	5571169	1175141	PRJNA680537	SRR13419227
IKEI	Lakota	Expandingleaf_1	30561527	30103038	21704855	1401858	236179	5353619	1406527	PRJNA680537	SRR13419228
IKEM	Lakota	Swellingbud_1	35925521	35140806	25435288	1685272	267801	6301127	1451318	PRJNA680537	SRR13419229
IKER	Lakota	Dormantbud_2	34409339	33496364	21626690	2861065	203938	7760043	1044628	PRJNA680537	SRR13419230
IKEW	Lakota	Expandingleaf_2	39342484	38437170	26589948	1692836	354810	7337804	2461772	PRJNA680537	SRR13419231
IKFA	Lakota	Catkin_2	32895103	32228477	21443841	1330221	306615	6894455	2253345	PRJNA680537	SRR13419232
IKFE	Lakota	Dormantbud_3	32554576	31851515	20139740	3117318	187024	7493007	914426	PRJNA680537	SRR13419233
IKFI	Lakota	Expandingleaf_3	37099364	36493558	25987143	1547637	307361	6977060	1674357	PRJNA680537	SRR13419234
IKDM	Pawnee	Expandingleaf_1	33887643	33495687	23838304	2330946	206691	6547781	571965	PRJNA680537	SRR13422673
IKDS	Pawnee	Swellingbud_1	36211607	35727875	24536811	2539980	202845	7948268	499971	PRJNA680537	SRR13422674
IKDX	Pawnee	Catkin_1	33714954	33325730	21656548	2244771	194375	8780696	449340	PRJNA680537	SRR13422675
IKEB	Pawnee	Dormantbud_1	25551216	25229809	16470301	2635464	120689	5691040	312315	PRJNA680537	SRR13422676
IKEF	Pawnee	Expandingleaf_2	36618138	36193308	24117203	2360641	197522	8985885	532057	PRJNA680537	SRR13422677
IKEJ	Pawnee	Swellingbud_2	31280422	30895396	20606472	2170516	163865	7505136	449407	PRJNA680537	SRR13422678
IKEN	Pawnee	Catkin_2	36979548	36579904	24481535	2386465	140687	9152683	418534	PRJNA680537	SRR13422679
IKES	Pawnee	Dormantbud_2	37538543	37076220	24821739	4158913	146494	7503257	445817	PRJNA680537	SRR13422680
IKEX	Pawnee	Expandingleaf_3	30236521	29838951	20380895	1985092	138839	6909910	424215	PRJNA680537	SRR13422681
IKFB	Pawnee	Swellingbud_3	33297295	32928789	22964145	2095304	176357	7225488	467495	PRJNA680537	SRR13422682
IKFF	Pawnee	Catkin_3	37134916	36657478	24176703	2158420	259679	9383855	678821	PRJNA680537	SRR13422684
IKFJ	Pawnee	Dormantbud_3	37703282	37334858	24524021	4172036	147314	8006888	484599	PRJNA680537	SRR13422683

*The first three metadata columns give the unique library ID, genome ID and the tissue type/replicate for each library.

**The following seven counts columns provide the number of raw and prepped reads, and counts of gene-, no gene-, ambiguous-, un- and multi-mapping reads.

***The rightmost two columns give the SRA bioproject IDs and identifiers for the libraries deposited on NCBI.

Supplementary Table 2. Tabulation of types of PAV among genomes*.

Sequence similarity**	Gene score confidence***	Elliott	Lakota	Oaxaca	Pawnee
Absent	High	375	120	162	86
Absent	Low	1964	1506	1492	1359
Absent	Moderate	722	297	327	245
Diverged	High	62	70	85	51
Diverged	Low	755	730	845	774
Diverged	Moderate	188	179	230	125
Very Similar	High	452	452	533	350
Very Similar	Low	3461	3264	3050	3456
Very Similar	Moderate	1041	1015	105	911

*Present genes in orthogroups with presence-absence variation (PAV) were mapped against genome assemblies that were missing orthologous gene models (absences).

**The resulting alignments were categorized as 'very similar' (>99% sequence coverage, ≥95% sequence identity) 'diverged' (75-99% sequence coverage, 75-95% sequence identity) or 'absent' (0-75% sequence coverage or 0-75% sequence identity).

***Gene scores (see methods) were binned into three equally sized quantiles based on the distribution of all gene models across all genes, regardless of PAV status. In this case, the three gene score confidence bins were defined as 'high' (>1012.256), 'moderate' (1012.256 - 177.664) and 'low' confidence (<166.664). The tabulation of these scores are not equal in the table below as bins were calculated on the total gene score distribution and low-score genes were highly enriched in PAV genes. The counts of each pairwise combination of gene score confidence and sequence similarity bins between present genes and un-annotated syntenic sequence are presented for each genome in their respective columns.

Supplementary Table 3. Extent of large (≥500-SNP) introgressions into the four reference genomes.

Plant ID	Aqu**.	Cor.	Myr.	Pecan	A (%)	C (%)	M (%)	P (%)
**'Oaxaca'***	**20.57**	**0.6**	**0**	**612.68**	**3.23**	**0.09**	**0**	**96.21**
87MX3-2.4	23.54	0.66	0.22	610.26	3.7	0.1	0.03	95.83
87MX4-5.5	21.6	1.25	0.41	610.99	3.39	0.2	0.06	95.95
87MX5-1.7	14.56	1.26	0	619.15	2.29	0.2	0	97.23
**'Elliott'**	**10.84**	**0**	**2.7**	**620.66**	**1.7**	**0**	**0.42**	**97.46**
'Curtis'	19.32	0	2.15	613.73	3.03	0	0.34	96.38
'Kanza'	18.57	7.71	0	606.76	2.92	1.21	0	95.28
'Kennedy	14.25	0	0.9	620.68	2.24	0	0.14	97.47
**'Lakota'**	**18.38**	**1.41**	**0**	**615.09**	**2.89**	**0.22**	**0**	**96.59**
'Major'	23.22	8.66	0	603.56	3.65	1.36	0	94.78
'Mohawk'	2.72	0	0.79	631.24	0.43	0	0.12	99.13
'Moore'	17.9	0	0.38	617.02	2.81	0	0.06	96.89
'Osage'	18.7	7.52	0	608.21	2.94	1.18	0	95.51
**'Pawnee'**	**6.63**	**0**	**0**	**629.09**	**1.04**	**0**	**0**	**98.79**
'Randall'	11.18	0	0	624.38	1.76	0	0	98.05
'Starking Hardy Giant'	14.78	0	0	619.89	2.32	0	0	97.34
'Success'	2.53	0	0	633.12	0.4	0	0	99.42

*Bolded rows are the reference genomes and are accompanied by three or four related genotypes.
**The proportion of ancestry is specified for three outgroups (*C. aquatica* (Aqu., A), *C. cordiformis* (Cor., C), *C. myristiciformis* (Myr. M)) and *C. illinoinensis* (Pecan, P). The first four columns are total introgressed sequence (megabases) and the next four columns are percent of total sequence of each ancestry type.

Supplementary Table 4. Library metadata for scab susceptibility RNA-sequencing experiments.

Lib*	Condition	n.raw**	n.map	n.assign	n.ambig	n.multi	SRA Bioproject	SRA BioID
IXBR	Control	67716244	20827832	1785944	334980	573552	PRJNA680537	SRR13423202
IXBU	Control	21476723	6103173	528358	98713	162173	PRJNA680537	SRR13423203
IXBY	Control	54465250	18003882	1316066	307758	458928	PRJNA680537	SRR13423204
IXCK	Treatment	30083720	10105331	963322	167236	269528	PRJNA680537	SRR13423205
IXCN	Treatment	80778148	37052085	3544531	590398	988252	PRJNA680537	SRR13423207
IXCR	Treatment	76031911	33070812	2657013	518121	840419	PRJNA680537	SRR13423206

*The first three metadata columns give the unique library ID, genotype (genome) ID and the tissue type/replicate for each library.
**The following seven counts columns provide the number of raw and prepped reads, and counts gene-, no gene-, ambiguous-, un- and multi-mapping reads.

Supplementary Table 5. Gene ontology enrichment terms in the scab resistance experiment*.

Cat.	GO.ID	Term**	n. annotated	n. significant	n. expected	*P*-value	BH-adj. *P*-value	regulation
BP	GO:0009611	Response to wounding	249	12	2.4	0.0000057	0.0139764	down
BP	GO:0010185	Regulation of cellular defense response	10	3	0.1	0.0001	0.1226	down
BP	GO:0009733	Response to auxin	456	14	4.4	0.00015	0.1226	down
BP	GO:0009773	Photosynthetic electron transport photosys. I	18	3	0.17	0.00065	0.31876	down
BP	GO:0045490	Pectin catabolic process	18	3	0.17	0.00065	0.31876	down
BP	GO:0009409	Response to cold	435	12	4.19	0.0011	0.449533333	down
BP	GO:0010143	Cutin biosynthetic process	23	3	0.22	0.00136	0.476388571	down
BP	GO:0009753	Response to jasmonic acid	258	9	2.49	0.00158	0.48427	down
BP	GO:0015977	Carbon fixation	19	4	0.18	0.00245	0.595167273	down
BP	GO:0019725	Cellular homeostasis	356	6	3.43	0.00248	0.595167273	down
BP	GO:0055114	oxidation-reduction process	1937	36	18.68	0.00267	0.595167273	down
BP	GO:2000022	Reg. of jasmonic acid mediated signaling	30	3	0.29	0.00296	0.604826667	down
BP	GO:1901568	Fatty acid derivative metabolic process	49	3	0.47	0.00393	0.647328	down
BP	GO:0071398	Cellular response to fatty acid	10	2	0.1	0.00396	0.647328	down
BP	GO:0071277	Cellular response to calcium ion	10	2	0.1	0.00396	0.647328	down
BP	GO:0042135	Neurotransmitter catabolic process	11	2	0.11	0.0048	0.653866667	down
BP	GO:0009835	Fruit ripening	11	2	0.11	0.0048	0.653866667	down
BP	GO:0019253	Reductive pentose-phosphate cycle	11	2	0.11	0.0048	0.653866667	down
BP	GO:0006636	unsaturatedfattyacidbiosyntheticprocess	14	2	0.13	0.0078	0.911910476	down
BP	GO:0043693	Monoterpene biosynthetic process	14	2	0.13	0.0078	0.911910476	down
BP	GO:0010200	Response to chitin	127	5	1.22	0.00781	0.911910476	down
BP	GO:0042737	Drug catabolic process	154	8	1.48	0.00882	0.953081739	down
BP	GO:0009071	Serine family amino acid catabolic process	15	2	0.14	0.00894	0.953081739	down
BP	GO:0009695	Jasmonic acid biosynthetic process	46	3	0.44	0.00989	1	down
MF	GO:0020037	Heme binding	494	15	4.76	0.000097	0.077018	down
MF	GO:0016717	Oxidoreductase activity	19	3	0.18	0.00076	0.30172	down
MF	GO:0005506	Iron ion binding	462	12	4.45	0.00184	0.4764	down
MF	GO:0016168	Chlorophyll binding	31	3	0.3	0.00325	0.4764	down
MF	GO:0016762	xyloglucan:xyloglucosyl transferase activity	35	3	0.34	0.0046	0.4764	down
MF	GO:0034007	S-linaloolsynthase activity	11	2	0.11	0.0048	0.4764	down
MF	GO:0005504	Fatty acid binding	11	2	0.11	0.0048	0.4764	down
MF	GO:0019825	Oxygen binding	11	2	0.11	0.0048	0.4764	down
MF	GO:0004497	Monooxygenase activity	371	10	3.57	0.00543	0.479046667	down
MF	GO:0009815	1-aminocyclopropane-1-carboxylateoxidase	13	2	0.13	0.00672	0.485061818	down
MF	GO:0051185	Coenzyme transmembrane transporter activity	13	2	0.13	0.00672	0.485061818	down
MF	GO:0051213	Dioxygenase activity	136	5	1.31	0.00954	0.575082857	down
MF	GO:0045330	Aspartylesterase activity	46	3	0.44	0.00987	0.575082857	down
CC	GO:0098807	Chloroplast thylakoid membrane protein comp.	14	5	0.14	0.0000002	0.0000814	down
CC	GO:0009522	Photosystem I	42	5	0.43	0.000067	0.0136345	down
CC	GO:0009535	Chloroplast thylakoid membrane	365	16	3.74	0.00083	0.08954	down
CC	GO:0010319	stromule	42	4	0.43	0.00088	0.08954	down
CC	GO:0048046	apoplast	239	8	2.45	0.00331	0.269434	down
CC	GO:0010287	plastoglobule	69	4	0.71	0.00551	0.3327225	down
CC	GO:0005576	Extracellular region	1024	23	10.5	0.00605	0.3327225	down
CC	GO:0031410	Cytoplasmic vesicle	2977	43	30.52	0.00654	0.3327225	down
CC	GO:0005618	Cell wall	394	10	4.04	0.00797	0.360421111	down
BP	GO:0010200	Response to chitin	127	12	0.62	1.3E-12	3.1876E-09	up
BP	GO:0071456	Cellular response to hypoxia	249	7	1.22	0.00022	0.26972	up
BP	GO:0006468	Protein phosphorylation	1781	20	8.71	0.00035	0.286066667	up
BP	GO:0008152	Metabolic process	12751	74	62.38	0.00279	1	up
BP	GO:0000288	nuclear-transcribed mRNA catabolic process	17	2	0.08	0.00307	1	up
BP	GO:0002213	Defense response to insect	31	3	0.15	0.0046	1	up
BP	GO:0009409	Response to cold	435	7	2.13	0.00554	1	up
BP	GO:0009751	Response to salicylic acid	243	5	1.19	0.00688	1	up
BP	GO:0071248	Cellular response to metal ion	27	2	0.13	0.00768	1	up
MF	GO:0004713	Protein tyrosine kinase activity	1570	20	8.13	0.00016	0.09925	up
MF	GO:0003700	DNA-binding transcription factor activity	1625	20	8.41	0.00025	0.09925	up
MF	GO:0030247	Polysaccharide binding	150	5	0.78	0.00111	0.29378	up
MF	GO:0016760	cellulosesynthase(UDP-forming) activity	44	3	0.23	0.00154	0.30569	up
MF	GO:0043565	sequence-specific DNA binding	721	9	3.73	0.00811	0.951917778	up
MF	GO:0030145	Manganese ion binding	82	3	0.42	0.00891	0.951917778	up
MF	GO:0004674	Protein serine/threonine kinase activity	681	9	3.53	0.00901	0.951917778	up
CC	GO:0031410	Cytoplasmic vesicle	2977	22	11.98	0.0025	1	up
CC	GO:0009505	plant-type cell wall	171	4	0.69	0.005	1	up

*Only significant terms with raw two-sided Fisher's test *P*-values < 0.01 are presented.
**The first three columns give metadata for each GO term (category, ID, description) and columns 4-8 present the statistics for the enrichment test (n. genes annotated in each category, number in the tested group, number expected, Fisher's P-value and FDR-adjusted P-value. The last column gives the regulation (up/down) type and the list of all genes in the test group. This experiment was conducted once at a single time point.

Supplementary Table 6. Candidate gene data for the main QTL peak for *P. russellae* resistance*.

gene ID**	pangenomePos	primChr	primStart	primEnd	altChr	altStart	altEnd	percPepId	diseaseAnnot
CiLak.16G012100	40918	Chr16	1521681	1528125	scaffold_855	47793	50713	58.6206897	Not clearly disease associated
CiEll.16G013600	40920				scaffold_855	59574	61157		Not clearly disease associated
CiLak.16G013300	40931	Chr16	1642581	1645058	scaffold_33	19940	21770	7.93650794	Not clearly disease associated
CiPaw.16G014500	40933				scaffold_33	15229	15340		Not clearly disease associated
CiPaw.16G015100	40937				scaffold_33	95224	204094		Disease associated with LRR-motif
CiPaw.16G015000	40938				scaffold_33	54171	54600		Not clearly disease associated
CiPaw.16G015600	40939				scaffold_33	421216	421740		Disease associated with LRR-motif
CiPaw.16G015500	40941				scaffold_33	324446	327005		Disease associated
Caril.16G014100	40944				scaffold_33	285108	286117		Not clearly disease associated
Caril.16G014200	40947				scaffold_33	558779	559102		Disease associated
CiEll.16G015100	40948				scaffold_33	70139	494316		Disease associated with LRR-motif
CiEll.16G015200	40949				scaffold_33	298347	327816		Disease associated with LRR-motif
CiPaw.16G015700	40951				scaffold_33	422605	422945		Not clearly disease associated
CiPaw.16G015800	40953				scaffold_33	424493	424680		Disease associated
CiLak.16G013900	40954	Chr16	1781863	1790118	scaffold_33	377203	379702	68.1701031	Disease associated with LRR-motif
CiLak.16G013800	40955	Chr16	1752618	1758703	scaffold_33	176923	177078	96.0784314	Disease associated
CiLak.16G014000	40956	Chr16	1790136	1795683	scaffold_33	342578	383674	94.4347826	Disease associated with LRR-motif
CiEll.16G015800	40957				scaffold_33	325929	326568		Disease associated with LRR-motif
CiEll.16G015900	40958				scaffold_33	409248	409463		Not clearly disease associated
CiEll.16G016000	40959				scaffold_33	410871	411305		Disease associated with LRR-motif
CiPaw.16G016000	40960				scaffold_33	438901	439433		Disease associated
CiLak.16G014100	40961	Chr16	1798540	1804542	scaffold_33	424823	430716	45.3125	Disease associated with LRR-motif
CiEll.16G016300	40962				scaffold_33	425645	428106		Disease associated with LRR-motif
CiLak.16G014200	40963	Chr16	1804973	1807424	scaffold_33	432294	432995	94.6308725	Not clearly disease associated
CiEll.16G016900	40964				scaffold_33	339692	340036		Disease associated
CiEll.16G017200	40966				scaffold_33	372165	372644		Not clearly disease associated
CiLak.16G014400	40967	Chr16	1821334	1823940	scaffold_33	597948	598427	93.7106918	Not clearly disease associated
Caril.16G015600	40969				scaffold_33	622971	625912		Disease associated with LRR-motif
CiPaw.16G016400	40970				scaffold_33	615353	616148		Not clearly disease associated
CiLak.16G014900	40973	Chr16	1849478	1853295	scaffold_33	663368	666773	93.6007641	Disease associated with LRR-motif
CiLak.16G018000	41009	Chr16	2208285	2211830	scaffold_4225	40996	44565	71.4285714	Disease associated
Caril.16G019100	41012				scaffold_4225	26371	27058		Not clearly disease associated
CiPaw.16G020100	41022				scaffold_4989	25351	31496		Not clearly disease associated
CiLak.16G019000	41025	Chr16	2387765	2392040	scaffold_4989	16128	31466	22.4489796	Not clearly disease associated

*We defined 34 total candidate genes in the region, 22 of which were only found on the alternative scaffolds and 12 where found as 1-to-1 orthologs between the two sequences but had significant peptide divergence (< 98% similarity).

**The gene ID and pangenome position are presented in the first two columns, while primary and alternative scaffold coordinates of all candidate genes are presented in columns 3-8. For 1-to-1 orthologs, the percent peptide identity is presented in column 9. The rightmost column contains a manual curation of genes in the interval, specifying whether annotations of homologs in other species are disease associated or similar to an annotated Leucine Rich Repeat (LRR) gene.

Supplementary Table 7. DNA resequencing library metadata for genotypes used in introgression analysis.

Lib*	PlantID	species	grp**	N bases***	Cov (X)	aligned_reads	mapped_bases	Map(%)	SRA Run	SRA Accession
IXDC	02_AQU_LA_BL1	C. aquatica	Caquatica	3.6899E+10	57	206174400	548003584	81	SRR14065918	PRJNA689620
IXID	92_AQU_TX_2	C. aquatica	Caquatica	4.3604E+10	67	231438952	550933979	78	SRR13361463	PRJNA689620
IXDB	02_AQU_LA_BF1	C. aquatica	Caquatica	4.3709E+10	67	241548178	553310225	81	SRR13361464	PRJNA689620
IXSD	02_COR_LA_BF_2	C. cordiformis	Ccordiformis	4.2121E+10	65	197685840	467071899	68	SRR13370650	PRJNA689620
IXIH	02_COR_LA_BF1	C. cordiformis	Ccordiformis	4.0412E+10	62	199956970	469240866	72	SRR13370651	PRJNA689620
IYAS	Randall	Pecan	elliott	2.6741E+10	41	154220910	592868768	83	SRR13521713	PRJNA680537
IYBA	StarkingHardyGiant	Pecan	pawnee	2.788E+10	43	163896290	593691228	85	SRR13521714	PRJNA680537
IXZY	Kennedy	Pecan	elliott	2.8957E+10	45	166675262	592622420	83	SRR13521711	PRJNA680537
IXKW	Moore	Pecan	elliott	2.9855E+10	46	170643868	593143136	83	SRR13521706	PRJNA680537
IWSA	Comanche	Pecan	truePecan	3.0529E+10	47	176535428	597360462	85	SRR13521699	PRJNA680537
IXDS	87_MX_5_1.7	Pecan	oaxaca	3.1067E+10	48	185397478	604552507	87	SRR13521705	PRJNA680537
IXZK	GraZona	Pecan	truePecan	3.2323E+10	50	188658162	598429575	84	SRR13521710	PRJNA680537
IXDR	87_MX_4_5.5	Pecan	oaxaca	3.2341E+10	50	192893952	606105269	87	SRR13521704	PRJNA680537
IXWU	Spence	Pecan	truePecan	3.3876E+10	52	193466494	595864842	83	SRR13521708	PRJNA680537
IYAC	Limpia_Creek	Pecan	truePecan	3.3719E+10	52	198018044	601661665	85	SRR13521712	PRJNA680537
IWWM	Mohawk	Pecan	pawnee	3.8229E+10	59	213254820	596840521	82	SRR13521700	PRJNA680537
IXDM	87_MX_3_2.4	Pecan	oaxaca	3.4743E+10	53	213939294	627922405	90	SRR13521703	PRJNA680537
IXYW	Curtis	Pecan	elliott	3.6505E+10	56	214153468	599653948	85	SRR13521709	PRJNA680537
IXTE	Burkett	Pecan	truePecan	3.9254E+10	60	225722918	604099642	83	SRR13521707	PRJNA680537
IYBB	Success	Pecan	pawnee	4.4789E+10	69	260797734	597464842	84	SRR13521716	PRJNA680537
IWWS	Kanza	Pecan	lakota	2.4812E+10	38	146145368	592142158	86	SRR13521702	PRJNA680537
IWWP	Osage	Pecan	lakota	2.5831E+10	40	149852624	595924743	85	SRR13521701	PRJNA680537
IYBQ	Major	Pecan	lakota	4.2539E+10	65	246994736	577223146	84	SRR13521715	PRJNA680537
IXLQ	07_MYR_LA_1	C. myrsiciformis	Cmyrsiciformis	2.8305E+10	44	143826334	448754565	74	SRR13370926	PRJNA689620
IXLR	08_MYR_AL_1.5	C. myrsiciformis	Cmyrsiciformis	3.063E+10	47	154626122	452820690	74	SRR13370927	PRJNA689620
IYBZ	92_MYR_TX_1G	C. myrsiciformis	Cmyrsiciformis	3.7031E+10	57	186828348	463177141	73	SRR13370928	PRJNA689620
IIMT	87MX3	Pecan	oaxaca	1.39E+11	214	731167006	641833840	79	SRR13430833	PRJNA680537
IKFU	LAKOTA	Pecan	lakota	6.0885E+10	94	311693958	605255273	77	SRR13403762	PRJNA680537
IPZM	ELLIOTT	Pecan	elliott	1.25E+11	193	640294398	612044251	76	SRR13403763	PRJNA680537
JAED	PAWNEE	Pecan	pawnee	9.1529E+10	141	450284968	610613427	71	SRR13404306	PRJNA680537

*The first three columns give the unique library IDs, breeding pedigree cultivar/genotype IDs and species IDs.

**The fifth column gives the experimental classification for the hidden Markov model (HMM), where the training libraries are given as their species IDs (or 'true pecan' for *C. illinoinensis* training libraries), and test libraries are specified by their focal reference genotype.

***The following five columns give the total number of sequenced bases, genomic coverage, total number of aligned reads, number of reads constrained to coverage between 8×-500× and the percentage of reads mapping. The rightmost two columns give the SRA run and accession identifiers for the libraries deposited on NCBI.
