# Supplementary material for: Four chromosome scale genomes and a pan-genome annotation to accelerate pecan tree breeding
Source: Nat Commun. 2021 Jul 5;12:4125. doi: 10.1038/s41467-021-24328-w (PMC8257795; doi:10.1038/s41467-021-24328-w)
Supplement: Supplementary file 8 — Description of Additional Supplementary Files [file 41467_2021_24328_MOESM8_ESM.docx]

Description of additional supplementary files

Title: Supplementary Data 1.

Description: Synteny-constrained pan-genome, restricted to subgraphs (orthogroups) present in at least one of the pecan genomes. The first two columns give the pan-genome coordinates of each subgraph. The next two columns give the representative genome and gene ID for each subgraph. The following five columns provide the gene IDs for members of each subgraph for each of the five species. If a gene is absent for a given genome, that cell is left blank. In cases where multiple genes are present in a subgraph within a genome, gene IDs are ‘|’ separated.

Title: Supplementary Data 2.

Description: Candidate genes within high-confidence introgression regions. For each non-pecan introgression (rows) in Figure 2B the coordinates and genes therein are listed. The coordinates and genome ID of the introgressions are provided in columns 1-5. Column 6 gives a “|” separated list of all genes physically within the introgression coordinates.

Title: Supplementary Data 3.

Description: DESeq2-derived differential expression statistics for all genes in the ‘Pawnee’ genome. Each row (gene) presents Wald-test contrast statistics (mean of base group, log2 fold-change, standard error of log2 fold-change, t-statistic, two-sided P-value and FDR-corrected P-value) between the inoculated and control groups. This experiment was conducted once at a single time point.

Title: Supplementary Data 4.

Description: Library metadata including SRA identifiers for short read libraries used to generate the ‘Oaxaca’ × ‘Lakota’ F1 genetic map. The first five columns give various genotype identifiers including the BioSample and NCBI SRA IDs. Columns 6-8 give library depth and coverage. The last three columns provide phenotype information and the mostly likely genotype at the Chr16 QTL peak.

Title: Supplementary Data 5.

Description: The F1 genetic map used for QTL mapping. The 11,489 ancestry informative SNP loci are presented (rows). The first three columns give the SNP ID, chromosome and physical position of the variant. The fourth column provides the genetic mapping position of each marker. Alleles for ‘Oaxaca’, ‘Lakota’ and the ‘Lakota’ parents (where available) are presented in columns 5-9 along with the marker phase in column 10. The rightmost column gives the LOD score from the one-way QTL analysis for phylloxera rating. This experiment was conducted once at a single time point.
