# Supplementary material for: Four chromosome scale genomes and a pan-genome annotation to accelerate pecan tree breeding
Source: Nat Commun. 2021 Jul 5;12:4125. doi: 10.1038/s41467-021-24328-w (PMC8257795; doi:10.1038/s41467-021-24328-w)
Supplement: Supplementary file 9 — Reporting Summary [file 41467_2021_24328_MOESM9_ESM.pdf]

nature research

Corresponding author(s): John Lovell, Jeremy Schmutz, Jennifer Randall

Last updated by author(s): Jun 1, 2021

Reporting Summary

Nature Research wishes to improve the reproducibility of the work that we publish. This form provides structure for consistency and transparency in reporting. For further information on Nature Research policies, see our Editorial Policies and the Editorial Policy Checklist.

Statistics

For all statistical analyses, confirm that the following items are present in the figure legend, table legend, main text, or Methods section.

n/a	Confirmed	
☐	☒	The exact sample size (*n*) for each experimental group/condition, given as a discrete number and unit of measurement
☒	☐	A statement on whether measurements were taken from distinct samples or whether the same sample was measured repeatedly
☐	☒	The statistical test(s) used AND whether they are one- or two-sided<br>*Only common tests should be described solely by name; describe more complex techniques in the Methods section.*
☐	☒	A description of all covariates tested
☐	☒	A description of any assumptions or corrections, such as tests of normality and adjustment for multiple comparisons
☐	☒	A full description of the statistical parameters including central tendency (e.g. means) or other basic estimates (e.g. regression coefficient) AND variation (e.g. standard deviation) or associated estimates of uncertainty (e.g. confidence intervals)
☐	☒	For null hypothesis testing, the test statistic (e.g. *F*, *t*, *r*) with confidence intervals, effect sizes, degrees of freedom and *P* value noted<br>*Give P values as exact values whenever suitable.*
☒	☐	For Bayesian analysis, information on the choice of priors and Markov chain Monte Carlo settings
☒	☐	For hierarchical and complex designs, identification of the appropriate level for tests and full reporting of outcomes
☒	☐	Estimates of effect sizes (e.g. Cohen's *d*, Pearson's *r*), indicating how they were calculated

Our web collection on statistics for biologists contains articles on many of the points above.

Software and code

Policy information about availability of computer code

Data collection	DNA and RNA data were collected via Illumina and PacBio internal routines. All phenotype data was collected and input manually using best practices for quantitative genetics data. No third party software was used for data collection
Data analysis	All data analysis was conducted through programs described in the methods. The majority of which was accomplished in the R environment for statistical computing. Other programs included: Ancestry_HMM v0.94, bcftools v1.9, samtools v1.9, varscan v2.4.3, bwa-mem v0.7.12, Picard v2.19.0, mafft v7.470, orthofinder v2.3.11, Repeatmasker v4.1.0, RepeatModeler v2.0.1, PASA v2.0.2, EXONERATE v2.4.0, MECAT v1, BLAT v36, QUIVERv2.2.2, HifiAsm v0.5, RACON v0.5, JUICER v1.5.6, diamond v0.9.36, orthofinder v2.3.11, MCScanX[no version], dbscan v1.1-5, MAFFT v7.470, Seqinr 4.2-5, gmap v2020-06-30, CLC v12.0.2, topGO v2.40.0, HTseq v0.9.1, STAR v2.7, Bowtie2 v2.3.4.1, Trimmomatic v0.36, FastQC v0.11.8, FGENESH v3.1.1, FGENESH_EST v2.6, GenomeScan v1, GENESPACE[no version].

For manuscripts utilizing custom algorithms or software that are central to the research but not yet described in published literature, software must be made available to editors and reviewers. We strongly encourage code deposition in a community repository (e.g. GitHub). See the Nature Research guidelines for submitting code & software for further information.

Data

Policy information about availability of data

All manuscripts must include a data availability statement. This statement should provide the following information, where applicable:

- Accession codes, unique identifiers, or web links for publicly available datasets
- A list of figures that have associated raw data
- A description of any restrictions on data availability

Genome assembly and annotation can be accessed at Phytozome and have been deposited in GenBank under BioProjects PRJNA680555 [https://www.ncbi.nlm.nih.gov/bioproject/PRJNA680555] ('Oaxaca'), PRJNA680556 [https://www.ncbi.nlm.nih.gov/bioproject/PRJNA680556] ('Pawnee'), PRJNA680557

[https://www.ncbi.nlm.nih.gov/bioproject/PRJNA680557] ('Lakota'), and PRJNA680558 [https://www.ncbi.nlm.nih.gov/bioproject/PRJNA680558] ('Elliott'). Genomes and annotations are also available through phytozome: 'Pawnee'[https://phytozome-next.jgi.doe.gov/info/CillinoinensisPawnee_v1_1], 'Elliott' [https://phytozome-next.jgi.doe.gov/info/CillinoinensisElliott_v1_1], 'Lakota' [https://phytozome-next.jgi.doe.gov/info/CillinoinensisLakota_v1_1], and 'Oaxaca'[https://phytozome-next.jgi.doe.gov/info/Cillinoinensis_v1_1]. RNA sequencing reads for annotation and fungus-induced gene expression have been deposited under SRA BioProject PRJNA680537 [https://www.ncbi.nlm.nih.gov/bioproject/PRJNA680537]. See Supplementary Tables 1, 4 and 7 and Supplementary Data 4 for RNA and DNA resequencing short reads SRA identifiers. Resequencing reads for the 'Lakota' × 'Oaxaca' genetic map were deposited on SRA under BioProject number PRJNA679828 [https://www.ncbi.nlm.nih.gov/bioproject/PRJNA679828]. Data supporting the findings of this work are available within the paper and its Supplementary Information files: synteny-constrained pan-genome (Supplementary Data 1), candidate genes within introgressions (Data 2), differential expression statistics and GO terms for response to V. effusa (Data 3 and Supplementary Table 5), Phylloxera QTL alleles, statistics and candidate genes (Data 4, Data 5 and Supplementary Table 6), and gene presence-absence summaries (Supplementary Table 2). A reporting summary for this article is available as a Supplementary Information file. Source data are provided with this paper either as a excel notebook (Figs. 1, 2a-c and Supplementary Figs. 1b, and 2), or in the Supplementary Data and Tables (Figs. 2d, 3, Supplementary Figs. 4). Additional datasets analyzed during the current study are available from the corresponding authors (jschmutz@hudsonalpha.org or jlovell@hudsonalpha.org) upon request.

Field-specific reporting

Please select the one below that is the best fit for your research. If you are not sure, read the appropriate sections before making your selection.

☒ Life sciences ☐ Behavioural & social sciences ☐ Ecological, evolutionary & environmental sciences

For a reference copy of the document with all sections, see nature.com/documents/nr-reporting-summary-flat.pdf

Life sciences study design

All studies must disclose on these points even when the disclosure is negative.

Sample size	No statistical tests were used to determine sample sizes. The number of genomes (4) was limited by funding. The number of resequenced genotypes (3-4 / reference genome) was chosen as the number required to make any statistical inference (3) and the number of related genotypes available. The number of RNA samples for annotation was the maximum we were able to sequence under our budget and covered all major tissues available. The number of biological replicates in the differential expression experiment was set to 3 as this is the minimum required for reasonable inference via linear modeling. The number of F1 progeny (143) was the maximum we could sequence under the budget.
Data exclusions	No data was excluded
Replication	No studies were re-conducted to test repeatability.
Randomization	Where appropriate, field and lab experiments employed a completely randomized design. Samples were randomly assigned experimental groups
Blinding	Investigators were not aware of genotype identifiers while conducting experiments or sequencing. Data analysis was not blind to experimental groups as these are the factors in the statistical test.

Reporting for specific materials, systems and methods

We require information from authors about some types of materials, experimental systems and methods used in many studies. Here, indicate whether each material, system or method listed is relevant to your study. If you are not sure if a list item applies to your research, read the appropriate section before selecting a response.

Materials & experimental systems

n/a	Involved in the study	
☒	☐	Antibodies
☒	☐	Eukaryotic cell lines
☒	☐	Palaeontology and archaeology
☒	☐	Animals and other organisms
☒	☐	Human research participants
☒	☐	Clinical data
☒	☐	Dual use research of concern

Methods

n/a	Involved in the study	
☒	☐	ChIP-seq
☒	☐	Flow cytometry
☒	☐	MRI-based neuroimaging
